# Crowdsourcing to identify social innovation initiatives in health in low- and middle-income countries

**DOI:** 10.1186/s40249-020-00751-x

**Published:** 2020-10-07

**Authors:** Lindi van Niekerk, Arturo Ongkeko, Rachel Alice Hounsell, Barwani Khaura Msiska, Don Pascal Mathanga, Josselyn Mothe, Noel Juban, Phyllis Awor, Dina Balabanova

**Affiliations:** 1 Graduate School of Business, University of Cape Town, Cape Town, South Africa; 2 London School of Hygiene and Tropical Medicine, London, UK; 3 College of Medicine / National Institutes of Health, University of the Philippines Manila, Manila, Philippines; 4 College of Medicine, University of Malawi, Blantyre, Malawi; 5 Centro Internacional de Entrenamiento e Investigaciones Medicas (CIDEIM), Cali, Colombia; 6 Pan American Health Organization, Washington, D.C USA; 7 School of Public Health, Makerere University, Kampala, Uganda

**Keywords:** Social innovation, Health, Crowdsourcing, Grassroots initiatives, Participatory research

## Abstract

**Background:**

Crowdsourcing is a distributed problem-solving and production mechanism that leverages the collective intelligence of non-expert individuals and networked communities for specific goals. Social innovation (SI) initiatives aim to address health challenges in a sustainable manner, with a potential to strengthen health systems. They are developed by actors from different backgrounds and disciplines. This paper describes the application of crowdsourcing as a research method to explore SI initiatives in health.

**Methods:**

The study explored crowdsourcing as a method to identify SI initiatives implemented in Africa, Asia and Latin America. While crowdsourcing has been used in high-income country settings, there is limited knowledge on its use, benefits and challenges in low- and middle-income country (LMIC) settings. From 2014 to 2018, six crowdsourcing contests were conducted at global, regional and national levels.

**Results:**

A total of 305 eligible projects were identified; of these 38 SI initiatives in health were identified. We describe the process used to perform a crowdsourcing contest for SI, the outcome of the contests, and the challenges and opportunities when using this mechanism in LMICs.

**Conclusions:**

We demonstrate that crowdsourcing is a participatory method, that is able to identify bottom-up or grassroots SI initiatives developed by non-traditional actors.

## Background

Crowdsourcing is an “online, distributed, problem-solving, and production model that uses the collective intelligence of networked communities for specific purposes” [1]. As a problem-solving model, crowdsourcing leverages collective wisdom by including people across different disciplines, sectors and organisations [2]. At the core of the approach is a strong belief that community members and citizens can provide answers to complicated problems for which solutions are not yet available. Crowdsourcing combines top-down, traditional project management with bottom-up, open innovation principles. Crowdsourced solutions are often proposed by those with direct experience of the challenge, and thus are grounded in the realities of everyday life [3, 4]. By using crowdsourcing contests, solutions to specific challenges can be harnessed by voluntary contributions from a wide range of actors.

The use of crowdsourcing has been growing in science-based and health fields. Yet, this approach has mainly been applied in high-income countries (HICs) and there is a lack of application in low - and middle-income countries (LMICs). This is substantiated in a systematic review by Ranard et al. [5] that described 21 studies in health using crowdsourcing, and of these, the majority were in the United States (*n* = 18) and Canada (*n* = 1). Within a LMIC context, crowdsourcing was applied in only two studies, one from India and one from South Africa [5]. In a more recent review by Crequit et al. [6], 202 studies in various health research fields used crowdsourcing as an approach. Similarly, this was mainly done in the United States, Canada, and Australia. The authors further note that key steps, characteristics, and logistics of crowdsourcing contests were poorly reported, thus limiting its quality and replicability. In the health sector, crowdsourcing has been applied in the fields of public health (health promotion) and in psychiatry, surgery and oncology [6]. It has been used as a way to support different tasks: problem-solving, data processing, surveillance and surveying, and clinical guideline development [3]. The benefits of crowdsourcing for global health research has been described as reducing data collection costs, while increasing sample size of participants, collecting information rapidly, and giving researchers access to data in real time [6, 7]. The majority of studies applying crowdsourcing in health used online technology platforms to facilitate the process. This, in itself, could be a reason why crowdsourcing has not been applied to the same extent in LMICs, where often investment in new technology and internet infrastructure is more limited.

Social innovation (SI) has emerged as a way of understanding and effecting lasting social change, especially when current systems and structures are failing those they are intended to serve. It is considered to be especially appropriate and effective to meet the social needs and advance the social wellbeing of people who are vulnerable or excluded [8–10]. SI initiatives can be regarded as “transformations in complex adaptive systems,” shifting the institutional and structural dimensions of the systems and, in so doing, generating resilience [11]. These initiatives can take multiple forms — it can be a product, process, service, policy or market mechanisms [8].

SI differs from technical innovations in four main ways. Firstly, the intended result of SI is to generate social impact rather than profit [8, 9]. Secondly, while technical innovations are primarily directed at scientific advancement, SI are orientated towards institutionalising new social practices [12–14]. Thirdly, SI are less concerned with novelty and result from combining existing or new elements from different organizations, disciplines and sectors [13]. Lastly, SI extends beyond invention to include implementation and capacity building of all participating actors and institutions and is embedded in the realities and dynamics of local health system and social contexts [11, 15]. In Table 1, we summarise the core qualities of a SI.

**Table 1:** Qualities of social innovation initiatives

Needs-based	Social innovations are explicitly designed to meet the contextual and social needs of the people or its intended user in order to improve their quality of life or wellbeing [13, 16–19].
Bottom-up participation	Social innovations are co-created and implemented through participation by different actors from the bottom-up and across disciplinary or organisational boundaries [20–25].
Qualities	Social innovations are more effective, efficient, sustainable or just than existing solutions [19].
Capacitating	Social innovations empower people to create new roles, establish relationships and develop assets and capabilities for better utilisation of resources [26].
Systems changing	Social innovations change the institutional and structural arrangements within established systems [27, 28].

The majority of evidence found in published literature on SI in health are from a HIC context and there is a dearth of published evidence from LMIC context. In a HIC context, SI typically address specific context-bound health or social challenges. The scarcity of documented examples in LMICs could be due to: firstly, a limited awareness of concept of social innovation in LMIC contexts. Our premise was that not only do SI initiatives exist at grassroots level, but implementers do not regard themselves as social innovators and neither is it a concept well recognised by academia nor policy-makers. Secondly, there is often a lack of research engagement by the academic community in LMICs on SI in health – both in terms of identifying these initiatives and studying their effects. These reasons hinder evidence from being generated and in turn, prevents countries from leveraging the potential benefits that adopting or scaling successful SIs could hold as part of policy and practice agendas to strengthen health systems [11, 29, 30]. Thus, we postulated that in a LMIC context, given the existence of an even greater array of social and health system challenges, it is likely that SI initiatives will exist in order to fill important gaps.

Given this gap in the literature, we sought to identify SI initiatives in health, in Africa, Asia and Latin America, and in so doing attempt to narrow the evidence gap on SI in health implemented in LMICs. This study focused specifically on SI in health in order to stimulate more research to be conducted and greater awareness to be raised about this concept.

In this article, we describe crowdsourcing as a method to identify SIs in health in LMICs. This approach offers an incentive for participation and a reward for those selected, while assisting the identification of SI initiatives not otherwise not known to the public health research community. We address the challenges and limitations of each step of crowdsourcing and discuss the applicability of the approach for its wider application in research in LMIC settings.

## Methods

### Rationale for crowdsourcing

To identify SI initiatives in health, crowdsourcing was selected as a method of choice for three reasons. Firstly, literature showed that SI initiatives are often developed by actors who operate outside the formal health system such as citizens, entrepreneurs or civil society organisations. These initiatives are often enabled by informal social networks and relationships. SI initiatives are many a time small-scale and unusual, and because they span sectors and social spaces, they tend to be less recognised by the health system. In addition, these implementing actors may lack the ability to conduct and publish research on their initiatives and therefore many LMICs health researchers have yet to engage in this field of inquiry. For these reasons, there are a limited number of publications to be found in peer-reviewed literature. Crowdsourcing as a method and process is aligned with the bottom-up, inclusive nature of SI, and gives equal opportunity for participation to a diverse range of actors, including non-traditional actors operating in health.

Secondly, conventional research approaches are often limited in scope and ability to reach large groups of people simultaneously and in a cost-effective manner. Crowdsourcing has the ability to reach multiple actor groups simultaneously. Thirdly, SI initiatives are durable and sustainable. With this as a key characteristic, it excludes many research projects described in published literature. Research initiatives are usually funded for a specific duration of time and once results are available, the projects are finalised, and implementation ceased. Thus, in order to find initiatives that are on-going and sustainable, a literature review would not yield sufficient results.

### Crowdsourcing contests in LMICs

From 2014 to 2018, six crowdsourcing innovation contests were conducted in three rounds, at global, regional and national levels. Each round assisted in identifying SI initiatives that are locally designed and implemented by country actors. The location and geographic focus of each of the crowdsourcing contests were purposefully selected, based on the availability and interest of a university or research centre (see Table 2). Universities who participated in this research project were all part of a larger research initiative — the Social Innovation in Health Initiative (SIHI), initiated by the Special Programme of Research and Training in Tropical Diseases (TDR) in 2014. As part of SIHI, participating universities received research capacity building and funding to identify, study and advocate for SI in health in their respective contexts.

**Table 2:** Contest overview

Round	Year	Implementer/ Social Innovation Research Hub	Health Challenge/s	Geographic Focus	Applications Received	Eligible Applications	Social Innovations Selected
1. Global South	2014–2015	University of Cape Town in partnership with Oxford University and the Special Programme for Research and Training in Tropical Disease.	Globally, 500 000 people die each year from Neglected Tropical Diseases (NTD) and 1 billion people are affected (WHO) [24]. 400 million people around the world do not have access to essential health services [31].	Africa	101	88	14
				Asia	50	50	7
				Latin America	12	12	2
2. Regional	2017	CIDEIM, in partnership with ICESI University, the Pan American Health Organization and the London School of Hygiene and Tropical Medicine	The burden of neglected tropical diseases in Latin America and the Caribbean (LAC) comprises 8.8% of the total global burden of disease [32].	Latin America & the Caribbean	16	7	3
3. National	2017	University of the Philippines, in partnership with the Philippines Department of Health and LSHTM.	About 60% of the Filipinos die without seeing a doctor [33]. There are about 77 000 adults and children living with HIV. In 2018, UNAIDS estimates showed that there is a 174% increase in the new HIV infections [34]. Around 1 M Filipinos are expected to have Tuberculosis and may or may not even know it [35].	Philippines	17	6	4
	2017	Makerere University in partnership with the Ugandan Ministry of Health and LSHTM.	The maternal mortality ratio in Uganda is 343 maternal deaths per every 100 000 live births; and the under-five mortality rate is 55 child deaths per 1000 live births [36].	Uganda	51	21	5
	2017	University of Malawi, in partnership with the Malawian Ministry of Health and LSHTM.	One in every 37 children in Malawi dies in the first month of life. For every 1000 live births in Malawi between 4 and 5 women die during pregnancy, childbirth, or within 42 days after childbirth. One in 8 children die from preventable diseases such as malaria, pneumonia and diarrhea [37].	Malawi	18	11	1
	2018	University of Malawi, in partnership with the Malawian Ministry of Health and LSHTM.	A significant proportion of the Malawian population is still underserved: 24% do not have access to a health facility within 5-kms and over 50% of leading causes of deaths are preventable [37].	Malawi	25	20	2
**Total**					**305**	**225**	**38**

*CIDEIM* Centro Internacional de Entrenamiento e Investigaciones Médicas*, ICESI* Instituto Colombiano de Estudios Superiores de Incolda, *LAC* Latin America and the Caribbean, *LSHTM* London School of Hygiene and Tropical Medicine, *HIV* Human immunodeficiency virus, *UNAIDS* Joint United Nations Programme on HIV/AIDS

The participating universities included: the University of Cape Town’s Bertha Centre for Social Innovation and Entrepreneurship; the University of Oxford’s Skoll Centre; the London School of Hygiene and Tropical Medicine, the University of Malawi, College of Medicine; Makerere University School of Public Health; University of the Philippines Manila, School of Medicine; and Centro Internacional de Entrenamiento e Investigaciones Médicas (CIDEIM) collaborating with ICESI University and the Pan American Health Organization (PAHO).

The countries selected for implementation of the various crowdsourcing contests were based on the participating university’s geographic focus area. The country crowdsourcing contests in Malawi, Uganda, the Philippines and Latin America region received technical assistance from London School of Hygiene and Tropical Medicine, as part of the collaborative consortium sub-grant.

## Results

**Fig. 1:**
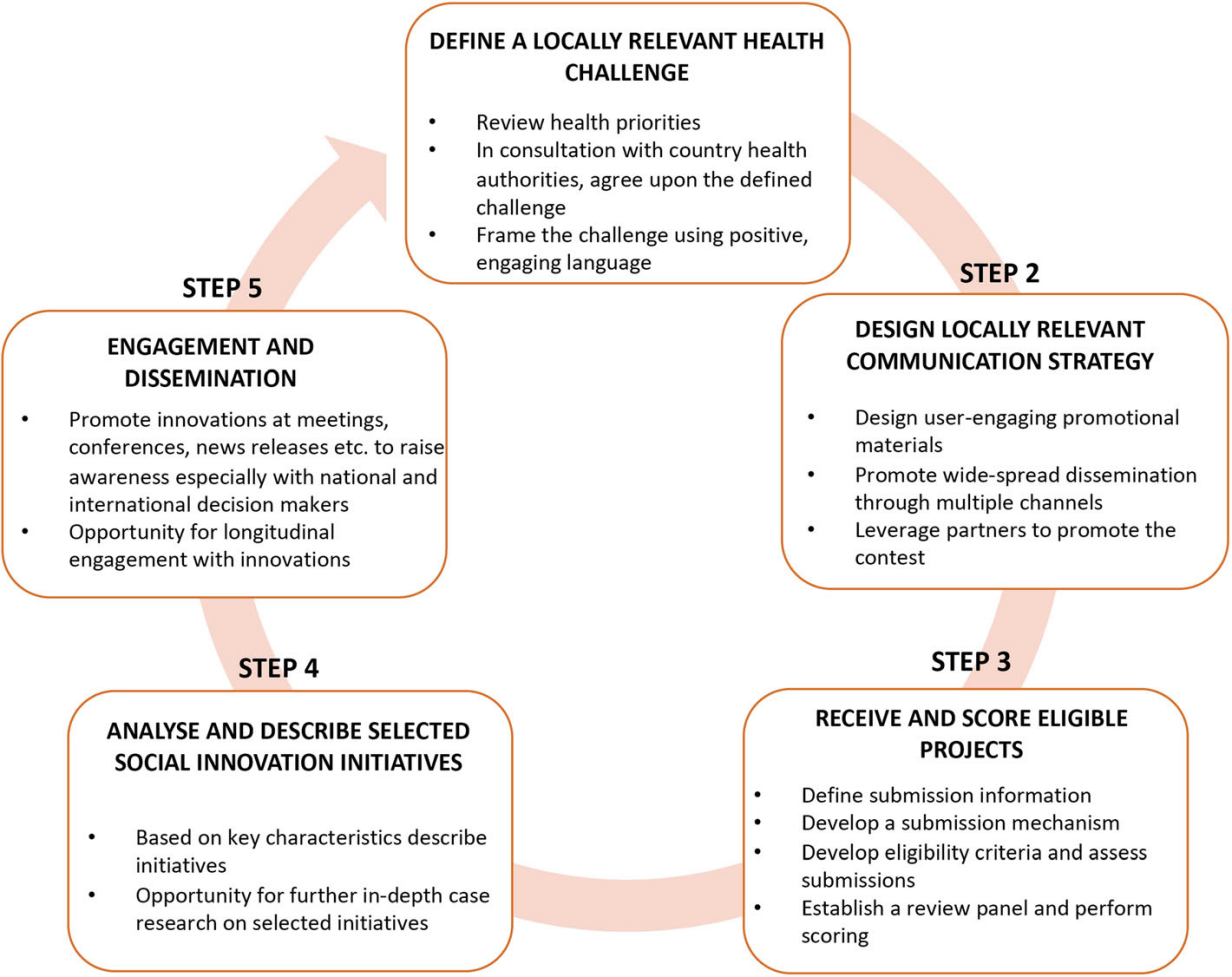
Crowdsourcing contest steps

Six crowdsourcing contests were implemented in a standardised process, consisting of five steps, across all participating institutions and countries. These steps were: 1. Define the locally relevant innovation challenge; 2. Design locally relevant communication strategies and promote the contest; 3. Receive and score eligible projects; 4. Analyse and describe potential SI initiatives in health; and 5. Engagement and dissemination (see Fig. 1).

To coordinate and maintain the standardised approach across each contest, several measures were implemented. Weekly teleconferences were held in the lead up to, and throughout the duration of, the contest to address any concerns, and provide support for the implementation of the standardised steps. The same infrastructure for the contests was used throughout (e.g. a central web portal for submissions, identical submission forms, templates for each step). At the conclusion of each contest, meetings with all the teams were held to reflect on the process.

### Step 1: Define the locally relevant challenge

For each crowdsourcing contest, a relevant challenge, in line with a key health priority, was defined and then shared with the public to request potential solutions that had already been developed to address this challenge. The challenge sought to provide a sufficiently clear focus, but not to be overly narrow and specific as to limit the type of solutions being put forth.

For the global contest, the challenge was defined through consultation with TDR, hosted at the World Health Organization, and at regional level in Latin America, through consultation with the PAHO, based on key priorities in LMICs of neglected tropical diseases and primary health service delivery. At national level, each implementing partner defined the challenge through consultation with their National Ministry of Health, based on key national priorities (see Table 2).

Stakeholder consultation on the challenge ensured that it was aligned to a global, regional or national priority. Consultation with these institutions enhanced their ownership of the contest and their support during the selection process, while fostering interest in the uptake of the SI initiatives identified (see Step 5).

The request put forward to potential applicants was to share any creative solution they had developed and implemented in response to the challenge area in LMICs or select countries. As SI is not a well-described concept in LMICs, and because people often do not qualify their own work as innovative, but rather, a simple solution to a given problem, the words ‘social innovation’ and ‘innovation’ were used sparingly and instead, the words ‘creative solutions’ were used. At the same time, it was acknowledged that any terminology, especially with translation, carries a risk of bias in interpretation by potential applicants.

Innovation challenges framed broadly in non-medical terms — for example, “Have you developed a creative solution that has transformed healthcare for mothers and babies in Uganda?” — received higher participation rates with greater diversity of applicants as compared to challenges that were framed using more technical and medicalised language, such as in the Latin American and Caribbean regional contest.

### Step 2: Design locally relevant communication strategies and promote the contest

Prior to launching the crowdsourcing contest, a contest promotion strategy was defined, supported by various communication products. To reach the widest possible audience, a contact list was developed; this included all known health implementers in each setting including international agencies, government bodies, universities, not-for-profits, private organisations, innovation incubators, professional bodies and community groups. A communication schedule was developed to ensure that all organisations were reached while promoting the contest. The communication products included flyers, posters, a video promoting the contest, videos sharing examples of social innovations (where available), and a social media campaign leveraging Facebook, Twitter, and LinkedIn. The package was tailored to be geographically and culturally representative to each setting using representative images and was translated into the main local languages. Context specific strategies were adopted to disseminate the communication products (see Table 3).

**Table 3:** Communication strategies

	Call location of focus	Main promotion strategies used
1	Global: Africa, Asia, Latin America and the Caribbean	Newsletters communication to 3000 individuals across all regions Personalised emails Telephonic interviews to individuals in key global health organisations
2	Regional: Latin America and the Caribbean	Email communication to 450 organisations across 20 countries E-News communication of partner organisations e.g. PAHO, USAID Social media: Facebook, Twitter, Linked In
3	National: Philippines	Presentations at key health events e.g. National health research forum Personalised to email communication to 78 organised & personal connection Promotion via partners – University of the Philippines Manila and Philippine Council for Health Research & Development Community online news channels e.g. Rappler Social media: Facebook & Twitter Radio
4	National: Uganda	Newspaper advertisements Radio advertisements Print posters and flyers WhatsApp communication to 120 district health officers Promotional video
5	National: Malawi (2017)	Newsletters Email communication Facebook Press briefing Promotional video
6	National: Malawi (2018)	Personalised communication with known individuals working in innovation / key health organisations Newspaper advertisements Press briefing Television features (prime time) Radio features Engaged existing organisations with a strong community presence to spread the message through their network. WhatsApp messaging and Facebook Promotional video

*PAHO* Pan-American Health Organization, *USAID* United States Agency for International Development

Each innovation contest solicited applications for 6 weeks, with active dissemination and promotion of the contest prior to and during this period. For these contests, no monetary prize was offered; rather, the ‘prize’ was recognition and showcasing at a national or international level to decision-makers and funders.

The effectiveness of the approach and the number of submissions received were heavily dependent on the extent to which the contests were promoted and disseminated across countries and districts. The presence of existing social networks and internet connectivity were two factors influencing the number of applications received.

In the 2014–2015 contest, the implementing universities had stronger existing networks in Africa and Asia which supported the contest promotion as compared to the Latin America and Caribbean (LAC) region, and thus in fewer applications received from LAC. Similarly, in the case of the Malawian contests, a higher number was received during the 2018 contest as compared to the 2017 contest. In 2017, SI was a new concept in Malawi as well as crowdsourcing. With a limited number of pre-existing partners to support the SI crowdsourcing contest, who could leverage their own networks for promotion and dissemination, only a few applications were received. By 2018, the Malawi team established in-country partnerships that led to the broader recognition and promotion of SI and in so doing contributing to an increased number of applications compared to 2017. In all country settings, support from the National Ministry of Health, local partners or regional institutions such as PAHO, and their representation on all communication materials, assisted in the crowdsourcing contest gaining credibility and legitimacy.

The extent of internet connectivity also influenced the uptake of the crowdsourcing contest in LMICs. In the 2017, the promotion of the Malawi contest relied mainly on electronic promotion strategies (email, social media, e-newsletters). Due to high data costs and limited internet connectivity, the Malawian population is less active on social media and other electronic platforms (2.7% of the Malawian population is active on Facebook [38], compared with 71% of the population in the Philippines [39]). Thus, in 2018, the contest in Malawi was repeated using different communication approaches, one which relied more on radio, television, community structures and in-person meetings to promote the contest. This increased the number of applications received from across the country.

From the outset, it was decided to provide only non-financial rewards as an incentive for the contest. For more established organisations and actors, receiving recognition, sharing their work and making a global contribution was a sufficient reward for their participation. Yet for smaller organisations the lack of a financial award could have contributed to their reduced participation (as indicated by applicant queries). Financial incentives hold much greater value for smaller SI initiatives while public recognition is of greater value to larger more established SI initiatives.

### Step 3: Receive and score eligible projects

A survey application form was designed to capture all relevant information from applicants. The survey form was made available via a purposefully designed online application portal. Each applicant received notification of receipt, giving them a unique identification number and confirming the next steps in the process.

Once the contest deadline was reached, all survey forms that had been submitted were screened for eligibility by the implementation team. In addition to the specific health challenge defined for each contest (described in Table 2), the following criteria, shared publicly, were applied when screening each application for eligibility:

- The solution is developed by any person (irrespective of disciplinary background), any organisational structure (public, private), non-government organizations (NGOs) or community group.
- The solution is inclusive (able to reach many people), effective (shown impact, or potential to make an impact) and affordable (as compared to the current standard).
- The solution is implemented and operational in the specified geographic area.
- The solution has been operational for at least 1 year.
- The solution cannot be a medical, scientific solution or advanced device innovation

In 2014–2015, the following weighted criteria were used to further assess the eligible applications (see Table 4), based on core characteristics of social innovation as defined in literature.

**Table 4:** Global and regional criteria

Criteria	Description	Weight (%)
Appropriateness of the solution to the need	The approach addresses a health-care delivery challenge that specifically deals with an infectious disease of poverty or could be applicable to this disease group	10
Degree of innovativeness	The approach is new, different or a significant improvement within the context to which it is being applied	25
Inclusiveness	The approach has the potential to be used by many people, enhancing equity and access	15
Affordability	The solution is affordable to the poor who are otherwise excluded in the local context or the solution is more cost-effective than the status quo	10
Effectiveness	The solution has a demonstrated positive outcome on the health of the local population	15
Scalable	Within and across cultural, resource and environmental contexts, the solution can be applied to reach many more people	10
Sustainable	The financial, organizational and market aspects of the solution are sustainable	15

In 2017–2018, the criteria were refined to allow for greater country participation and ownership (see Table 5). Four criteria remained constant, but each implementer was given the opportunity to co-create three additional criteria of value and relevance to their country context or national priorities. Potential criteria were solicited from each independent review panel member. The most commonly proposed criteria were included in the final criteria.

**Table 5:** National contest criteria

	Criteria	Description	Weight (%)
**Baseline criteria**	Degree of innovativeness	The approach is new, different or a significant improvement within the context to which it is being applied	15
	Inclusiveness	The approach has the potential to be used by many people, enhancing equity and access	15
	Affordability	The solution is affordable to the poor who are otherwise excluded in the local context or the solution is more cost-effective than the status quo	15
	Effectiveness	The solution has a demonstrated positive outcome on the health of the local population	15
*Above plus 3-country/region-specific criteria: Each independent review panel identified four additional selection criteria that were country-specific based on national priorities, weighted at 10% each*			30
**National Criteria**	Philippines	The solution addresses a health priority of the Philippines (as defined by the National Unified Health Research Agenda), or a priority in a more localized level such as prevalent yet neglected health problem in a town or a marginalised group/ethnic group Participatory approach is evident in the development, implementation, and evaluation of the innovation (i.e. contributions from various stakeholders: the patients/families, local health personnel, local leaders, other sectors). Feasibility for the solution to be applied, replicated and scaled-up to other communities with similar problems.	
	Uganda	A good understanding of the problem itself, the people most affected and the size of the problem so as to warrant large scale involvement of the various stakeholders from the social innovation circles. A well thought-through sustainability model that details integration into existing programmes or work processes. Does it have the potential to be scaled up and can it be widely accepted in our context?	
	Malawi	The solution fulfils a practical need in the healthy sector and / or meets a particular problem in a community The solution is user-friendly to the community i.e. it is uncomplicated to use and does not require sophisticated training. The solution has a possibility to be sustained and demonstrates that they have considered issues to ensure long-term sustainability.	

For each contest, the implementing team established an independent external review panel of between 8 and 20 members. Panellists were known experts in each region or country with experience in public or global health, respective challenge area, health systems, clinical care, innovation and SI. In addition, frontline clinicians and community members were included on each panel to ensure bottom-up views of the health system. The function of the panel was to review and score all eligible applications received.

Each eligible nomination was assigned to at least two members of the review panel, one purposefully assigned based on their own expertise and the application’s focus area, and one randomly assigned. Review panel members signed onto the online platform with their own unique login and password. An electronic audit trail documented the review process. Reviewers scored each selection criterion on a rating scale, with a score of 4.5–5 being outstanding and a score of 1 being flawed. The weights assigned to each selection criterion were standardised across the scoring sheet and applied automatically. The scores from each panel member who reviewed an application were averaged. The score had to be 3.5 or higher to be considered for final selection.

From the review panel members, a smaller core review panel was established. It functioned firstly, to assess any applications where the scores assigned by the review panel members differed by more than 40%, and secondly, to re-review the highest-scored applications to decide which had the greatest potential to be true SI initiatives, based on the self-reported data.

The application form used across all calls consisted of five sections gathering basic descriptive information on the implementing organisation, the creator, and the proposed project. In addition, narrative answers were requested on the extent of problem being addressed, the solution and its innovativeness, and the impact. In the first contest (2014–2015), the application forms were poorly completed. International organisations working in the respective countries were familiar with this application approach, but many smaller national level applicants lacked experience in completing applications of this nature. The application form was only distributed in English and Spanish, and failure to include local languages further hindered the quality of applications. The decision was therefore taken to give all applicants an opportunity to revise their application form based on standard application guidance. In subsequent contests, this guidance was included in the application form. Implementing partners also offered potential applicants assistance with completing the form if required.

The extent of information requested was to ensure that the independent review panel had enough content to guide their decision making. The unintended consequence was that applicants were deterred due to the length and complexity of the process, and the time required to complete the form. Often applicants had to seek approval from the organisational leaders to apply, and this further delayed submission.

The review panel was established ahead of each contest and members’ time prioritised. Yet, panellists had competing time priorities, and the allocated time frame for the review process (3 weeks) was insufficient. Additionally, few review panellists had expert knowledge in SI in the context of LMICs. The scores thus did not always assist in distinguish between an SI initiative and a ‘good public health’ intervention. Although independent review panels provide credibility and legitimacy to the contest, the value of the review panel in identifying and selecting SI initiatives may be limited.

Receiving the application form via an online portal assisted in ensuring that all data were in a single repository and that these could be readily accessed by the review panel, from any location. However, due to data costs and limited, unpredictable internet connectivity in some contest countries, the portal was not always accessible. Paper-based application forms were provided as an option to applicants and on receipt, the implementation team entered the form contents onto the online portal.

### Step 4: Analyse and describe potential social innovations in health

The study team analysed all submitted data to extract relevant characteristics. Given that data is self-reported, its accuracy and quality cannot be verified through crowdsourcing contests alone. For the 38 potential social innovations selected through the review process, field visits were conducted to each site and qualitative data were gathered to verify the project as a SI initiative, and to derive potential lessons and learning relevant to health systems. Case studies were produced on each of the 38 SI initiatives [17].

From the six crowdsourcing contests conducted 2014–2018, a total of 38 SI initiatives were identified (see Table 6). The majority of these initiatives (47%) were created and implemented by not-for-profit, non-governmental organisations or social enterprise organisations, with half operating within the Africa region. The main focus (66%) was to fill a service delivery gap within the existing health system, primarily by providing primary care services at community level. The innovating or founding actors had a variety of backgrounds as health care professionals, business entrepreneurs, public health researchers, scientists, parliamentary members, engineers and community members. The average duration for which these innovations had been active in implementing their work (as of 2019) was 10.7 years.

**Table 6:** Characteristics of the 38 Selected Social Innovations by region (percentage of total)

		Africa	Asia	Latin America	Total
**Organisational Structure**	For Profit	18%	18%	0%	**16%**
	Not for Profit / NGO / Social Enterprise	59%	28%	40%	**47%**
	Government Institution	0%	36%	0%	**11%**
	University	14%	18%	60%	**21%**
	Partnership project (several entities involved)	9%	0%	0%	**5%**
**Location of implementation**	Community-based Delivery	50%	45%	80%	**53%**
	Facility-based Delivery	36%	45%	0%	**34%**
	Community-facility Linkage	14%	10%	20%	**13%**
**Actors engaged in delivery**	Formal health care worker (doctor, nurse, public health official)	64%	64%	60%	**63%**
	Community or family member	23%	18%	20%	**21%**
	Community health worker	14%	18%	20%	**16%**
**Main Programme Focus (*not mutually exclusive*)**	Training / Education	50%	45%	80%	**53%**
	Service Delivery	82%	45%	40%	**66%**
	Community Mobilisation	23%	36%	40%	**29%**
	Technology	32%	45%	40%	**37%**
	Research	9%	36%	60%	**24%**
**Partnership with Government**	Yes	73%	82%	60%	**74%**
	No	27%	18%	40%	**26%**
**Innovator background**	Medical professional	45%	27%	20%	**37%**
	Business entrepreneur	23%	27%	0%	**21%**
	Public Health Researcher	14%	0%	60%	**16%**
	Scientist	0%	19%	20%	**8%**
	Other	18%	27%	0%	**18%**
**Innovator Gender**	Female	41%	45%	80%	**47%**
	Male	59%	55%	20%	**53%**
**Innovator Nationality**	LMIC	55%	82%	100%	**68%**
	HIC	45%	18%	0%	**32%**

*NGO* Non-government organization, *LMIC* low – and – middle income countries, *HIC* high – income countries

The crowdsourcing contests provided information on existing programmes that were not possible to access through the literature (published or grey). Several did not have their own website. Encouraging citizens across 48 countries to participate in the contest revealed many unknown and non-traditional actors across sectors participating in different ways to improve health in LMICs.

### Step 5: Engagement and dissemination

As data collection was not anonymous and applications were made for the purpose of being known and recognised, implementing partners had the opportunity to provide feedback to all applicants and to continue ongoing discussions with each of them. The outcome of each call was disseminated at national, regional and international level through the key organisations and individuals involved in the process.

This engagement led to case study research being conducted on each SI initiative, thus supporting a possible strong future relationship. Since the crowdsourcing contests and field visits, implementing partners have been able to track and monitor the evolution of several SI initiatives, to deepen and extend the inquiry through ongoing data collection, and to continue comparing and contrasting social innovations across different settings. The SI implementers have continued to provide and share new information with the research teams, giving researchers’ opportunities to deepen the case research and showcase the SI initiatives. SI implementers have gained greater insight into the value of research as well as received opportunities to form new connections, networks and tap into funding opportunities. During the field visits in 2015, two selected SI initiatives were excluded as sufficient data had not been collected.

At a national level, through early engagement with the Ministry of Health, policy makers in Malawi, Uganda and the Philippines were able to learn from and leverage locally developed bottom-up SI initiatives. At least 50% or more of these initiatives were not known to policy makers.

## Discussion

This research was intended to scope SI initiatives in health using crowdsourcing as a methodology to solicit applications from a range of implementers and researchers in LMICs; across Africa, Asia and Latin America. The value of crowdsourcing as an approach is that aligns with the bottom-up, participatory and inclusive nature of SI. It has been well described in the literature and its potential contribution to public health has been shared [8, 15, 19, 40]. However, its application has been limited mainly in HICs settings with limited application in LMIC settings. Through six crowdsourcing contests conducted at a global, regional and national level between 2014 and 2018, a total of 305 eligible projects were identified, and of these 38 were selected as strong case examples of SI initiatives.

At a national level, the crowdsourcing contests extended the awareness of locally driven health initiatives and allowed the participating universities to actively engage with new actors such as community-based groups, NGOs or social enterprises. Through further analysis and in-depth case study research, new lessons were described that have sparked the interest of national Ministries of Health and local development partners. The crowdsourcing contests provided a form of credibility to participating universities as knowledgeable experts in SI in their country [41].

From experience gained in implementing these contests, we have several recommendations for future contests in LMICs. The innovation challenge needs to be framed in broad, non-medicalised and positive way around a health challenge to encourage participation. By engaging the relevant agency or ministry in selecting the challenge focus, country ownership, uptake and dissemination are enhanced. Ample time investment and resources are required to prepare and promote the contest, and without these, the outcome of the contest may be limited. Contest promotion in LMICs needs to use more diverse means, for example supplementing electronic communication with personal and other communication strategies, and leveraging already existing platforms. Establishing partnerships with key health implementing organisations in the country is an effective way to increase the reach and promote the contests.

Reducing the complexity of the application process, in terms of the accessibility and the extent of information required, would increase participation, lighten the load on implementers and use available resources more effectively. For future contests, a two-step application process is recommended: the first step might be a simplified survey form, with an interim criterion applied to assess the merit of application and whether further information should be requested for the second stage.

The support of an independent review panel would be valuable during the first round of ‘screening’ and the speed by which the review is done would be enhanced with less content for review. A small panel of experts in SI would be valuable to assess the second stage of applications. Increased time in the contest cycle needs to be allocated for this process. If crowdsourcing contests are used as a methodology, follow-up research, further data collection and analytical steps are required to verify findings.

Several limitations of the analysis need to be acknowledged. The study involved a descriptive analysis of the crowdsourcing process conducted by the authors in several LMICs, making a case for using crowdsourcing as a novel approach to identify SI and other health initiatives. However, while the crowdsourcing process described in this paper offers detailed guidance on its implementation, there are debates regarding the advantages of different methodological steps [40], and it may not be generalizable to all LMICs contexts. Moreover, the study was retrospective, which may have introduced a degree of recall bias.

## Conclusions

Crowdsourcing contests offer an engaged participatory form of research, providing a range of non-traditional actors with a new opportunity to contribute and share data within health systems. This method enabled the identification of multiple SI initiatives that are effectively addressing health challenges in LMICs in creative ways, initiatives that otherwise would remain unknown to local and international decision-makers. Crowdsourcing offers a benefit to several other health system actors: researchers have the opportunity to gain a broad range of data in a cost-effective way and access new opportunities to conduct research in partnership with SI implementers; SI implementers receive recognition and in turn support global advocacy of the concept of SI as applied in health, and new evidence generated from further investigation of these SI initiatives could inform policy and practice in support of health systems strengthening in LMICs.

## Data Availability

Data sharing is not applicable to this article as no datasets were generated.

## Abbreviations

CIDEIM: Centro Internacional de Entrenamiento e Investigaciones Médicas
HIC: High-income countries
LMIC: Low- and middle-income countries
NGO: Non-governmental organisation
PAHO: Pan American Health Organization
SI: Social innovation
SIHI: Social Innovation in Health Initiative
TDR: Special Programme for Research and Training in Tropical Diseases, cosponsored by the United Nations Children’s Fund, the United Nations development Programme, the World Bank and the World Health Organization
